# The effect of clopidogrel on platelet activity in patients with and without type-2 diabetes mellitus: a comparative study

**DOI:** 10.1186/s12933-015-0182-7

**Published:** 2015-02-03

**Authors:** Claudia Schuette, Daniel Steffens, Marco Witkowski, Caroline Stellbaum, Peter Bobbert, Heinz-Peter Schultheiss, Ursula Rauch

**Affiliations:** Department of Internal Medicine/Cardiology, Campus Benjamin Franklin, Charité - Universitätsmedizin Berlin, Hindenburgdamm 30, 12200 Berlin, Germany

**Keywords:** Clopidogrel, Diabetes, Platelet function, Ccoronary heart disease, Percutaneous coronary intervention

## Abstract

**Background:**

Although antiplatelet therapy involving clopidogrel is a standard treatment for preventing cardiovascular events after coronary stent implantation, patients can display differential responses. Here, we assessed the effectiveness of clopidogrel on platelet function inhibition in subjects with and without type-2 diabetes and stable coronary artery disease. In addition, we investigated the correlation between platelet function and routine clinical parameters.

**Methods:**

A total of 64 patients with stable coronary heart disease were enrolled in the study. Among these, 32 had known type-2 diabetes, whereas the remaining 32 subjects were non-diabetics (control group). A loading dose of 300 mg clopidogrel was given to clopidogrel-naïve patients (13 patients in the diabetes group and 14 control patients). All patients were given a daily maintenance dose of 75 mg clopidogrel. In addition, all patients received 100 mg ASA per day. Agonist-induced platelet aggregation measurements were performed on hirudin-anticoagulated blood using an impedance aggregometer (Multiple Platelet Function Analyzer, Dynabyte, Munich, Germany). Blood samples were drawn from the antecubital vein 24 h after coronary angiography with percutaneous coronary intervention. The platelets were then stimulated with ADP alone or ADP and prostaglandin-E (ADP and ADP-PGE tests, respectively) in order to evaluate clopidogrel-mediated inhibition of platelet function. The effectiveness of ASA was measured by stimulation with arachidonic acid (ASPI test). In addition, maximal platelet aggregation was assessed via stimulation with thrombin receptor-activating peptide (TRAP test).

**Results:**

Patients with diabetes exhibited significantly less inhibition of platelet function than patients without diabetes (ADP-PGE test p = 0.003; ASPI test p = 0.022). Administering a clopidogrel loading dose of 300 mg did not result in a lower level of ADP-PGE-induced platelet reactivity in comparison to the use of a 75 mg maintenance dose. Moreover, we observed that ADP-PGE-induced platelet inhibition was positively correlated with fasting blood glucose and HbA1c (p < 0.01).

**Conclusions:**

Patients with type-2 diabetes exhibited increased platelet reactivity compared to patients without diabetes despite combined treatment with clopidogrel and ASA. Using a loading dose of clopidogrel rather than small daily doses was not sufficient for adequately overcoming increased platelet reactivity in patients with type-2 diabetes, highlighting the need for more effective anti-platelet drugs for such patients.

## Background

Combined antiplatelet therapy using acetylsalicylic acid (ASA) and clopidogrel is the standard therapy regimen for secondary prevention of cardiovascular events after coronary stent implantation in patients with stable coronary artery disease [1-3]. However, variable inter- and intra-individual responses to clopidogrel (i.e., “clopidogrel low responsiveness”) represent a significant clinical limitation [4,5]. In addition, various drug interactions have been reported to affect clopidogrel-mediated inhibition of platelet function [6-8]. Notably, inefficient suppression of platelet activity upon treatment with clopidogrel and ASA has been associated with significantly increased cardiovascular risk [9,10].

In 2000, we reported that shear-stress-induced thrombus formation on the tunica media of an injured blood vessel was significantly increased when blood was obtained from patients with type-2 diabetes as compared to non-diabetics [11]. Moreover, we found that hyperglycemia and leukocyte count positively correlated with increased thrombus formation [11], and that intensive glycemic control in patients with type-2 diabetes was effective in decreasing blood thrombogenicity [12]. We also demonstrated that diabetic cardiovascular autonomic neuropathy and diabetic angiopathy were associated with increased platelet activation, possibly contributing to the pathogenesis of these conditions [13,14]. Additionally, it has previously been shown that mean platelet volume increases with increasing fasting blood glucose [15]. Thus, these alterations may collectively contribute to the association between poor glycemic control and poor outcome in patients undergoing percutaneous coronary intervention [16].

The response of patients with type-2 diabetes to anti-aggregatory therapy has therefore recently become an important topic of investigation. Indeed, Geisler et al. studied the effect of a 600 mg clopidogrel loading dose in patients with acute coronary syndrome with or without type-2 diabetes [17]. They reported that diabetic patients displayed a significantly higher risk for further atherothrombotic complications resulting from insufficient inhibition of platelet aggregation, and increased blood thrombogenicity. In addition, Angiolillo et al. found that clopidogrel withdrawal is associated with an increase in platelet and inflammatory biomarkers in diabetic patients [18].

In the present study, we examined the effect of combined treatment with clopidogrel and ASA on platelet function inhibition using impedance aggregometry. We compared agonist-induced platelet aggregation in patients with stable coronary artery disease with and without type-2 diabetes. Moreover, we evaluated the correlation between metabolic parameters and measures of platelet function.

## Methods

### Patients and anti-platelet medication strategies

**Figure 1:**
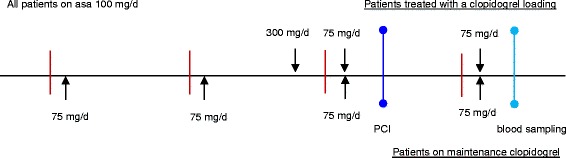
**Clinical study design.** Legend: Schematic representation of the study design. Patients presenting stable coronary heart disease with or without type-2 diabetes were enrolled. A 300 mg loading dose of clopidogrel was given to clopidogrel-naïve patients. All other patients were given a daily maintenance dose of 75 mg clopidogrel. In addition, all patients received acetylsalicylic acid (ASA; 100 mg/day). Blood samples were drawn 24 h after coronary angiography with percutaneous coronary intervention (PCI), and agonist-induced platelet aggregation measurements were performed.

This study included 64 consecutively enrolled patients with coronary heart disease and stable angina pectoris. Patients were included if they were aged 40 to 75 years, were receiving a statin along with ASA, and had been selected to undergo clopidogrel treatment at the discretion of their physician. Patients were excluded if they had experienced a myocardial infarction within the last 28 days; displayed elevation in troponin T or creatinine kinase; had been receiving long-term anticoagulation therapy; had known cancer, liver cirrhosis, or dialysis-dependent renal failure; were receiving antibiotic therapy; were pregnant; or were alcohol-dependent. Among the enrolled subjects, 32 patients had a confirmed diagnosis of type-2 diabetes and 32 patients were diabetes free. An oral glucose tolerance test was employed to exclude the presence of diabetes in the control group. The patient characteristics, concurrent medications, and laboratory blood test results are given in Table 1. All patients received 100 mg of ASA (uncoated) once daily. In the control group, 18 patients received 75 mg of clopidogrel once daily; in the diabetes group, 19 received 75 mg of clopidogrel once daily. The day prior to undergoing percutaneous coronary intervention (PCI), a 300 mg loading dose of clopidogrel was given to the 14 clopidogrel-naïve patients in the control group and the 13 in the diabetes group. Subsequently, all 64 patients were administered a maintenance dose of 75 mg clopidogrel per day. All patients received unfractionated heparin (UFH) during the catheterization. Platelet function was measured in all patients 24 h after coronary angiography and PCI (Figure 1). This study was approved by the local ethics committee, and all patients gave informed consent prior to study inclusion.

**Table 1:** **Patient characteristics**

	**Control**	**Diabetes**
	**(n = 32)**	**(n = 32)**
Age (y)	64.8 ± 12.5	70.2 ± 8.2
Sex (% women)	21.9	21.9
Platelets (nl)	248.5 ± 89.4	223.0 ± 75.4
BMI (kg/m^2^)	25.5 ± 3.2	30.0 ± 5.4
Leucocytes (nl)	6.9 ± 1.8	7.5 ± 2.5
HbA1c (%)	5.6 ± 0.3	7.2 ± 1.2
Fasting blood glucose (mg/dl)	84 ± 16.0	151 ± 33.1
CRP (mg/dl)	0.6 ± 1.5	0.8 ± 1.7
Cholesterol (mg/dl)	160 ± 33.5	157 ± 39.3
Triglycerides (mg/dl)	150 ± 69.9	165 ± 51.2
HDL-cholesterol (mg/dl)	45.8 ± 11.3	46.8 ± 18.9
LDL-cholesterol (mg/dl)	95.1 ± 24.1	98.2 ± 28.8
Concomitant drugs		
Statin (%)	96.9	100
CCB (%)	90.1	65.6
ASA-100 mg/d (%)	100	100
Antidiabetic drugs		
Metformin (%)	0	59.4
Insulin (%)	0	31.3
Other/none (%)	0	25.0
Clopidogrel		
Loading dose-300 mg (%)	43.8	40.6
Maintenance dose-75 mg/d (%)	100	100

Legend: BMI, body mass index; HbA1c, glycated hemoglobin; CRP, C-reactive protein; HDL, high-density lipoprotein; LDL, low-density lipoprotein; CCB, calcium channel blocker; ASA, acetylsalicylic acid.

### Assessing inhibition of platelet function

Venous blood was collected in test tubes containing hirudin (25 μg/ml), which allowed for inhibition of coagulation without affecting calcium levels. Platelet function was then analyzed using a Multiple Platelet Function Analyzer (Multiplate-Test, Dynabyte, Munich, Germany), which measures increases in impedance [19]. In brief, activated platelets adhere to sensor surfaces within the system and aggregate to form a barrier, which results in a constant increase in resistance between the electrodes. The extent of aggregation was measured over a period of 6 min and was calculated based on the area under the curve (AUC) [20]. Whole blood was first diluted with 0.9% NaCl at a ratio of 1:1 and then stored for 3 min in the test cell at 37°C. Subsequently, several reagents were added to induce platelet aggregation: 1) adenosine diphosphate (ADP, final concentration: 6.4 μmol/l) for assessment of ADP-dependent platelet aggregation; 2) ADP and prostaglandin-E (ADP-PGE, 6.4 μmol/l) for measuring the effectiveness of the P2Y12-receptor antagonist clopidogrel; 3) arachidonic acid (0.5 mmol/l, ASPI test) for evaluating the effectiveness of ASA on platelet function; and 4) thrombin receptor-activating peptide (TRAP; final concentration: 32 μmol/l) for stimulating thrombin receptors on platelets to induce maximal platelet aggregation.

### Definition of low responsiveness to clopidogrel

ADP-induced platelet aggregation as measured by impedance aggregometry has been shown to correlate well with that measured using the vasodilator-stimulated phosphoprotein (VASP) phosphorylation test, which is considered to be the gold standard for detecting clopidogrel low responders [21]. Based on this prior comparison by Stellbaum et al., we pre-defined clopidogrel low-responders as those with platelet aggregation above the 75th percentile (cut-off of 27 AUC for the ADP test or 10 AUC for the ADP-PGE test; data not shown).

### Statistical methods

As there were no comparable studies that could be used to obtain effect sizes at the time of designing the study, no power analysis for sample size calculation could be carried out. Therefore, we aimed to enroll at least n1 = n2 = 30 patients in both groups to ensure the proper conduct of parametric test statistics (i.e. robustness against violations of the assumptions of normality and variance homogeneity) to obtain the associated sensitivity.

To test for significant differences between the two groups a Student’s t-test for independent samples was used. In case of subsequent analyses with smaller and/or unequal sample sizes, we used the Mann–Whitney test statistic. To test for significant correlations, Pearson’s correlation coefficient was calculated and tested for significance as this test statistic reacts robustly to violations of the assumed bivariate normal distribution when tested against a null hypothesis of ρ = 0. All reported probability values are two-sided, and a value of p < 0.05 was considered to indicate statistical significance. Statistical analyses were carried out using SPSS software, version 16.0 for windows (SPSS, Inc., Chicago, IL, USA).

## Results

In the present study, we enrolled 64 patients with stable coronary heart disease. Among them, 32 had known type-2 diabetes, whereas the remaining 32 subjects were non-diabetics (control group). The characteristics of these patients are presented in Table 1.

**Figure 2:**
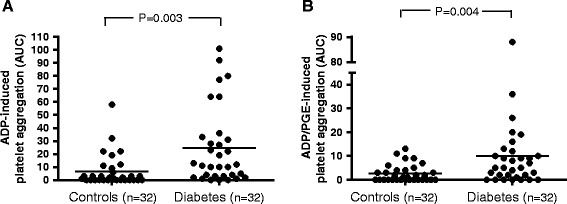
**ADP- and ADP-PGE -induced platelet aggregation in patients with and without diabetes.** Legend: Agonist-induced platelet aggregation measurements were performed on blood samples from diabetic and non-diabetic patients using an impedance aggregometer. The platelets were stimulated with adenosine diphosphate (ADP; **A**) alone or ADP and prostaglandin-E (ADP-PGE; **B**). P values were calculated using a student’s t-test for independent samples.

ADP- and ADP-PGE-induced platelet reactivity was found to be significantly higher in patients with type-2 diabetes compared to control patients, despite treatment with clopidogrel and ASA (Figure 2; ADP test: p = 0.003; ADP-PGE test: p = 0.004). Notably, 31% of the patients with type-2 diabetes exhibited ADP-induced platelet reactivity above 27 AUC, which was defined as clopidogrel low responsiveness. In contrast, only 6.4% of the patients without diabetes were classified as low responders (Figure 2A).

**Figure 3:**
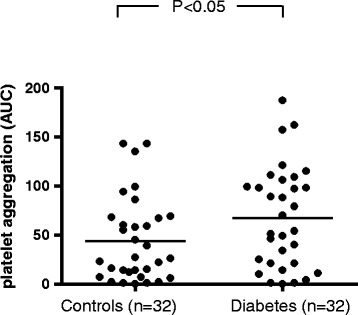
**TRAP-induced platelet aggregation in patients with and without diabetes.** Legend: Agonist-induced platelet aggregation measurements were performed on blood samples from diabetic and non-diabetic patients through impedance aggregometry. Maximal platelet aggregation was assessed via stimulation with thrombin receptor-activating peptide (TRAP). P values were calculated using a student’s t-test for independent samples.

**Figure 4:**
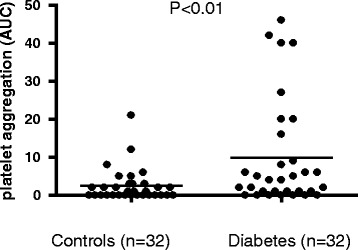
**ASPI test in patients with and without diabetes.** Legend: Platelet aggregation measurements were performed on blood samples from diabetic and non-diabetic patients using an impedance aggregometer. The effectiveness of acetylsalicylic acid (ASA) was measured by stimulation with arachidonic acid (ASPI test). P values were calculated using a student’s t-test for independent samples.

Considering TRAP-induced platelet reactivity, there was significantly higher platelet reactivity observed in the diabetes group when compared to non-diabetic patients (Figure 3, p < 0.05). In line with this, arachidonic acid-induced platelet reactivity (ASPI test) was significantly elevated in patients with diabetes (Figure 4, p = 0.01).

**Figure 5:**
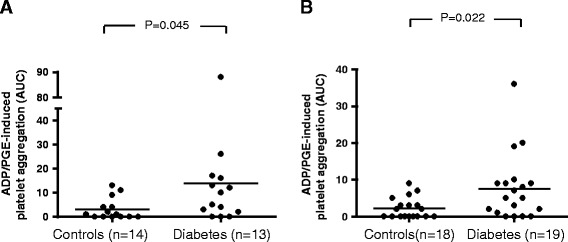
**ADP/PGE-induced platelet aggregation after clopidogrel loading and maintenance doses in diabetic and non-diabetic patients.** Legend: Agonist-induced platelet aggregation measurements were performed on blood samples through impedance aggregometry. The platelets were stimulated with adenosine diphosphate and prostaglandin (ADP-PGE) in order to evaluate clopidogrel-mediated inhibition of platelet function after a 300 mg loading dose **(A)**, and after a 75 mg maintenance dose **(B)** in patients with and without diabetes. P values were calculated using a student’s t-test for independent samples.

When comparing platelet aggregation in both groups after administration of a clopidogrel loading dose (300 mg), patients with type-2 diabetes continued to exhibit significantly higher ADP- and ADP-PGE-induced platelet reactivity compared to patients without diabetes (Figure 5A).

Finally, we observed that ADP-PGE-induced platelet aggregation positively correlated with fasting blood glucose (r = 0.37; p < 0.001) and HbA1c level (r = 0.36; p < 0.005).

## Discussion

Our findings indicate that combined treatment with clopidogrel and ASA is less effective in patients with type-2 diabetes than in those without. It is clear from the literature that there are many different factors that can have an effect on platelet reactivity in diabetic patients. Mortensen et al. found that diabetic patients with coronary heart disease displayed higher levels of sP-selectin than did non-diabetics, indicating increased platelet reactivity and higher cardiovascular risk [22]. A study by Erlinge et al. demonstrated that decreased response to clopidogrel by diabetic patients was likely due to a reduction in the amount of circulating active metabolite [23]. This indicates inefficient transformation of clopidogrel into the active metabolite in diabetics; however, the exact reason for this remains unclear. The increased cardiovascular risk in patients with type-2 diabetes has been shown to be related to higher levels of certain inflammatory markers [24,25]. Geisler et al. reported that diabetic patients with hyperglycemia had increased amounts of such markers in comparison to normoglycemics and non-diabetic patients [26]. Moreover, they showed that higher levels of inflammatory markers correlated with decreased response to ASA and clopidogrel dual therapy. Recently, Rosiak et al. reported decreases in a number of inflammatory markers in patients with type-2 diabetes on either raising the daily dosage of ASA or replacing ASA with clopidogrel [27]. Furthermore, they found that the effects were reduced in patients with poor long term glycemic control. Higher esterase activity has been identified in patients with type-2 diabetes in comparison to those without the condition. This has been speculated to be a cause of decreased efficacy of ASA therapy in diabetic patients [28]. Similarly, increased esterase activity in younger patients has been linked to reduced bioavailability of ASA, resulting in high platelet reactivity [29].

Although it is well accepted that platelet aggregation inhibition is necessary for patients with type-2 diabetes [30], there remains a need to develop more effective antiplatelet therapies [31]. Patients with diabetes are known to display increased populations of circulating platelets that express activation-dependent adhesion molecules that may contribute to increased aggregation and might provide new pharmacological targets [32]. Also, transient receptor potential canonical type 6 (TRPC6) calcium channels increase on platelets in response to high glucose [33], and could represent a novel target. Moreover, investigating the role of platelet-derived tissue factor-positive microparticles as well as adipose tissue-secreted hormones may be interesting with regard to therapeutic potential [34,35]. Indeed, leptin, resistin, and adiponectin have all been found to be increased in diabetic patients [35,36]. Thus, further research will be needed to assess these molecules as possible targets for novel antiplatelet therapies.

In addition, currently existing therapies could also be beneficial for diabetic patients. For example, we recently compared periprocedural platelet reactivity after administrating either bivalirudin, which directly binds to thrombin (the most effective activator of platelets), or unfractionated heparin during PCI, finding that bivalirudin was better than heparin at reducing platelet activation [37]. Thus, the efficacy of bivalirudin could be tested in diabetic patients and compared to current treatment approaches. Moreover, ADP receptor inhibitors (other than clopidogrel) have been assessed for use in patients with diabetes. Indeed, recent randomized studies demonstrated that ticagrelor was superior to prasugrel for reducing platelet reactivity in subjects with acute coronary syndrome and diabetes [38,39]. However, whether this higher potency of ticagrelor will translate into a clinical benefit for diabetic patients remains to be investigated. Nevertheless, it is interesting that treatment with ticagrelor led to a significant reduction in the rate of death from vascular causes, myocardial infarction, or stroke when compared to clopidogrel in acute coronary syndrome patients [40].

In the present study, we demonstrated that even a 300 mg loading dose of clopidogrel did not provide as effective platelet inhibition in patients with diabetes as that achieved in those without. Similar results were achieved by Angiolillo et al., who reported increased platelet reactivity in diabetic compared to non-diabetic patients, both after long term ASA/clopidogrel dual therapy and after a 300 mg loading dose, prior to PCI [31]. However, Sibbing et al. used a higher loading dose of 600 mg, and found no significant difference in platelet reactivity between diabetic and non-diabetic patients [41]. Thus, a significant proportion of patients with type-2 diabetes could display an insufficient therapeutic effect when treated with long term daily clopidogrel doses of 75 mg prior to PCI. A more recent study by Angiolillo et al. compared a 600 mg loading dose of clopidogrel to a 60 mg loading dose of prasugrel [42]. They found higher anti-platelet response in diabetic patients treated with prasugrel, in addition to a superior response profile. In combination, these results suggest that the use of alternative therapies such as ticagrelor or prasugrel could provide superior reductions in platelet reactivity in diabetic patients who do not respond sufficiently to clopidogrel.

Here, we observed that ADP-PGE-induced platelet aggregation positively correlated with fasting blood glucose and HbA1c. We have previously documented a correlation between thrombogenicity and blood glucose levels in type 2 diabetics [11]. Furthermore, we showed that a reduction in HbA1c level was associated with a reduction in blood thrombogenicity [12]. Angiolillo et al. additionally showed that patients with inefficiently regulated blood glucose exhibited a lower response to clopidogrel, along with increased blood thrombogenicity [31]. However, a significant relationship between ASPI-induced platelet aggregation and fasting blood glucose or HbA1c has not yet been reported. Thus, our results point to an association between actual glucose level and platelet reactivity, although further studies on a larger scale and including assessment of ASPI-induced aggregation are necessary before accurate conclusions can be drawn. Thus, glycemic control might favor a better response to anti-platelet therapies such as clopidogrel in patients with type-2 diabetes.

There are a number of limitations to this study. The low number of patients enrolled resulted in there being fewer than 20 in each of the four groups, with only 13 in the group consisting of diabetic patients receiving the clopidogrel loading dose. A further limitation is that platelet reactivity was not measured prior to initiating clopidogrel therapy. This was because patients were already receiving anticoagulation treatment with ASA.

## Conclusions

In summary, we have examined platelet activity utilizing impedance aggregometry, which represents a simple and fast method to assess inhibition of platelet function. Patients with type-2 diabetes exhibited an increased platelet reactivity compared to patients without diabetes, despite combined treatment with clopidogrel and ASA. Increasing clopidogrel dose was not sufficient for reducing the increased platelet reactivity in patients with type-2 diabetes, highlighting the need to further investigate other anti-platelet drugs in this population.

## Abbreviations

ADP: Adenosine diphosphate
ASA: Acetylsalicylic acid
AUC: Area under the curve
BMI: Body mass index
PGE: Prostaglandin-E
TRAP: Thrombin receptor-activating peptide
TRPC6: Transient receptor potential canonical type 6
VASP: Vasodilator-stimulated phosphoprotein
